# Selection of summer feeding sites and food resources by female migratory caribou (*Rangifer tarandus*) determined using camera collars

**DOI:** 10.1371/journal.pone.0294846

**Published:** 2023-11-29

**Authors:** Sophiane Béland, Barbara Vuillaume, Martin Leclerc, Martin Bernier, Steeve D. Côté

**Affiliations:** 1 Département de Biologie & Centre d’études Nordiques, Université Laval, Québec, Québec, Canada; 2 Département des Sciences Fondamentales & Centre d’étude de la Forêt, Université du Québec à Chicoutimi, Chicoutimi, Québec, Canada; 3 Département de Physique, de Génie Physique et d’optique, Université Laval, Québec, Québec, Canada; Universita degli Studi di Sassari, ITALY

## Abstract

Migratory caribou (*Rangifer tarandus)* is a socioeconomically and culturally key species for northern communities in the Arctic, and most of its populations are experiencing a sharp decline. Female migratory caribou depend on the availability of summer habitat resources to meet the needs associated with lactation and the accumulation of fat reserves to survive when resources are less abundant. Because of the large scales at which habitat and resource data are usually available, information on how female migratory caribou select habitat and resources at fine scales in the wild is lacking. To document selection of summer feeding sites, we equipped 60 female caribou with camera collars from 2016 to 2018. We collected a total of 65,150 10-sec videos between June 1^st^ and September 1^st^ for three years with contrasted spring phenology. We determined the selection at the feeding site scale (3^rd^ scale of Johnson) and food item scale (4^th^ scale of Johnson) using resource selection probability functions. Wetlands were highly selected as feeding sites in June and July while they were avoided in August. Shrublands were mostly selected in July and August. At the resources scale, lichen, birch, willow, and mushrooms were the most strongly selected resources. Our results provide precise and novel information on habitat selection at feeding sites and food resources selected by female caribou in the wild. This information will help understand foraging patterns and habitat selection behavior of female migratory caribou and will contribute to the management and conservation of its declining populations.

## Introduction

Understanding how animals adjust their behavior when facing different environmental conditions is of paramount importance in animal ecology. Habitat selection helps obtain fundamental information about the relation between an animal and its environment, and how an animal uses this environment and its components to survive, grow, and reproduce [1]. Habitat selection is defined as a behavioral process in which an individual utilizes resources in a non-random way, among the ones that are available [2–4]. Foraging behavior of an animal is dependent on multiple decisions, from the choice of the feeding site to the choice of which food item to eat [5–7]. To better identify scales at which selection occurs, Johnson [2] defined four orders. The first and second orders represent the geographical range and home range, whereas the third and fourth orders respectively represent selection of habitat components inside the home range (e.g., feeding sites) and the selection of items inside that habitat component, for example, food items within a feeding site. Habitat selection has been recognized as a scale sensitive process [8–10]. For example, Boyce et al. [11] found that elk (*Cervus canadensis*) selected for Douglas-fir forests at a broad scale (i.e., winter range), but were neutral in their selection and even avoided these forests at finer scales (i.e., home range and movement radius scale). Therefore, analyses at multiple spatial scales are preferable to better understand habitat selection, and results obtained at larger scales may not be transferable to smaller scales and vice versa [7, 10, 12].

Habitat selection is known to be influenced by biotic and abiotic factors, such as predation, landscape characteristics, weather, resource availability, or even insect harassment [13–16]. These factors can shape the selection behavior of animals at different spatial scales but can also influence multiple scales simultaneously [5]. For instance, Dussault et al [15] found that predation risk was the main driver of habitat selection by moose *(Alces alces)* at larger scales, whereas they had to trade-off predation risk and food availability at finer scales. On the contrary, Leclerc et al. [17] found no trade-off and female woodland caribou selected calving sites to reduce predation risk from large to fine scale habitat selection. Better understanding habitat selection decisions made by wildlife at multiple scales may help us better understand the limiting factors at play [5], which may be particularly relevant for declining populations.

Caribou and reindeer (*Rangifer tarandus*) have been facing strong declines across their circumpolar range in the last decades [18–20]. Due to their long-range movements and dispersion, caribou are usually studied at larger scales (i.e., first to third orders sensu Johnson [2]) with tools that collect data remotely or indirectly such as telemetry or indirect observations (e.g., tracks left in snow). Analysis of feces and stomachal content or observations on semi domesticated or tame animals are used for studying diet and food resources selection of caribou [6, 7, 21–24]. Studies from direct observations of resource acquisition of wild caribou are, however, nearly inexistent [21, 25–27]. Cameras mounted on satellite collars is a new technology that can help observing wild animal behavior directly at the finest scale of analysis, without any risk of disturbing them in the process [28, 29].

The Rivière-aux-Feuilles migratory caribou herd (RFH) in Québec (Canada) decreased from 628,000 individuals [30] in 2001 to 199,000 individuals in 2016 [20] (S1 Fig). Although the ecology of the herd is well studied, habitat selection of the RFH and any other herd of migratory caribou in summer, at a fine spatial scale is largely unknown. At a larger scale, using GPS data, Plante et al [31] found that in summer caribou of the RFH selected productive areas (i.e., higher normalized difference vegetation index (NDVI) values) with a higher abundance of shrublands and avoided areas with lichens or low vegetation. In the same herd, Brodeur et al. [32] also found that caribou, in summer, selected prostrate shrub tundra as well as erect-shrub tundra. A study by Crête, Huot and Gauthier [22] using rumen content showed that the June diet of RFH caribou consisted mostly of dead graminoids (49%) and lichens (25%), whereas in July, deciduous shrub leaves, lichens, and living graminoids made up 54, 11, and 10% of the diet, respectively. Using DNA barcoding from caribou pellets, Brodeur [32] also found that Ericaceae, Salicaceae, Mosses and Betulaceae dominated the caribou summer diet.

Using camera collars, we studied habitat selection of lactating female migratory caribou of the Rivière-aux-Feuilles herd in summer, at two increasingly fine scales: 1) the selection of habitat types as feeding sites within the home range of females (i.e., third-order selection, sensu Johnson [2]) and 2) the selection of food items within feeding sites (i.e., fourth-order selection, sensu Johnson [2]). We hypothesized that female caribou would select the most productive habitats as feeding sites to replenish their body reserves. We predicted that the selection for wetlands would decrease as the summer progresses while the selection for shrublands would increase. At the feeding site scale, we also assessed whether terrain ruggedness, water presence and insect harassment affected the feeding behavior of females. We hypothesized that caribou would modify their diet throughout the summer to track changes in vegetation quality. We predicted that they would select shrub leaves when they emerge, and that insect harassment would modulate their feeding behavior. With this study, we hoped to fill in the gaps in the knowledge about summer foraging patterns of migratory caribou at a fine scale.

## Methods

### Study area

The study area was located north of the 56^th^ parallel in northern Quebec, Canada. It encompassed the calving ground (~126,907 km^2^) and the summer range (~254,463 km^2^) of the RFH located west and northwest of the Ungava Bay in Nunavik. We calculated these ranges from 95% kernel based on GPS locations of the females from our study. The region is mainly covered by arctic tundra, dominated by shrubs (*Betula* sp., *Vaccinium* sp., and *Salix* sp.), terrestrial lichens, mosses, and graminoids [33]. The region also includes rocky areas, wet tundra dominated by carex and dwarf shrubs, and wetlands [33].

### Animal capture and data processing

From 2016 to 2018, we captured pregnant female caribou by net-gunning from a helicopter [34] and fitted them with camera-GPS collars (Vertex Plus, Vectronic Aerospace). We confirmed pregnancy with an echography test using an ultrasound scanner (ExaGo, ECM Noveko International Inc., Angoulême, France). Captures occurred at the end of March or beginning of April, which is at least 8 weeks before the beginning of the expected calving period. For the three years of the study (2016 to 2018), we deployed 14, 24, and 22 collars, respectively. Collars automatically detached each year in September, and we then retrieved them without the need of recapture. Because of collar malfunction (n = 5) or animal death (n = 3, died of natural causes > 2.5 months after capture), the final number of collared females was 14, 21, and 17. All capture and handling procedures were approved by the animal care committee of Université Laval and the Quebec Ministère des Forêts, de la Faune et des Parcs (MFFP).

Resolution of the cameras was 480p in 2016 and 2017, and in 2018, we also added four collars that were 720p. Each collar recorded 10-second videos every 20 minutes from 5:00 to 20:00 EST from 1 June to 1 September. We could only have one daily schedule for the entire summer period. At these latitudes, sunrise and sunset in August are about 05:00 and 20:00 EST, respectively. We therefore decided to use this schedule during the entire summer. Each collar collected an average of 3,870 videos for a total of 10 hours and 45 minutes of footage throughout the summer. We analyzed a third of the footage (1 video/hour) collected by each collar to limit the time required for video processing. To choose which video would be analyzed, we randomly selected the time of the first video analyzed for a day (8:00, 8:20 or 8:40) and then analyzed the videos recorded at hourly intervals. For example, if the time randomly selected for the first video was 8:40, then the second video analyzed was 9:40, then 10:40 and so on, for that day for that collar. We chose to do a randomized selection because we did not want to miss any behavior and could not know, a priori, whether any of the behaviors could be cyclic.

### Assessing use

We assessed caribou selection of feeding sites and food resources (i.e., third- and fourth-order selection, respectively; sensu Johnson [2]) by determining if a site or a resource type was used or not. At the feeding site scale, we considered a site used when an individual foraged at least once during a video. On the contrary, if an individual was exhibiting any other behavior (e.g., resting, walking, vigilance) without foraging, we considered this site to be unused. At the food resource scale, we only used videos where an individual was foraging, and we identified which food resources were consumed (i.e., used) and which could be seen on the video but not consumed (i.e., unused). To classify a food resource as consumed, the caribou needed to take a bite of the resource and chew it, determined by looking at movements from the mouth and throat. Each consumed resource was considered as used regardless of the number of bites taken, because it was often impossible to precisely count the number of bites taken from a specific resource. Consequently, in a video, at the feeding site, there could only be one type of habitat used or unused while for the food item scale, multiple food items could be used and unused.

### Data extraction from video

At the feeding site scale, we quantified the effect of habitat type, terrain ruggedness, abundance of flying insects, and the presence of water body on the probability of a caribou feeding at a certain site. For this purpose, we classified habitat types based on observation from video and with the help of the description of habitat types from the Northern Quebec Vegetation Map [35]. We combined and retained nine different habitat types for feeding sites (Table 1). Some habitat types were only observed during a specific period. For instance, snowy grounds were only present in June, while taiga was only encountered in August.

**Table 1:** Description of habitat types used to evaluate feeding site selection of female caribou of the Rivière-aux-Feuilles herd during summer in northern Québec, Canada.

Habitat Type	Description
Tundra	Vegetation composed of lichens, mosses, graminoids and/or herbaceous species. Fewer than 10% of trees (conifers).
Ericaceous tundra	Tundra vegetation with ericaceous shrubs (e.g., *Cassiope tetragona*; *Rhododendron groenlandicum*). Fewer than 10% of trees (conifers).
Tundra with shrubs	Tundra vegetation with birches and/or willows (less than 70%). Fewer than 10% of trees (conifers).
Wetland	Vegetation mostly composed of graminoids, lichens and mosses but can also include ericaceous and herbaceous species. Can be a stagnant body of water present all summer as well as a temporary body of water created by snow melting. Wet tundra is included in this category.
Wetland with shrubs	Wetland with birches and/or willows (less than 70%).
Shrubland	Vegetation composed of at least 70% of birches and/or willows. Can contain other types of vegetation under and around the shrubs.
Rocky ground	Area with rocks, rocky plateau or sandy area. Vegetation is sparse or inexistent. Lichens, graminoids or mosses are usually the only resource available.
Snowy ground	Area covered by snow. May have some sparse zones with vegetation poking out of snow. Only available in June.
Taiga	Treed area (conifers), with low vegetation composed of lichens (mostly *Cladonia* spp.), birches and/or willows, ericaceous and herbaceous species. Only used in August.

**Fig 1:**
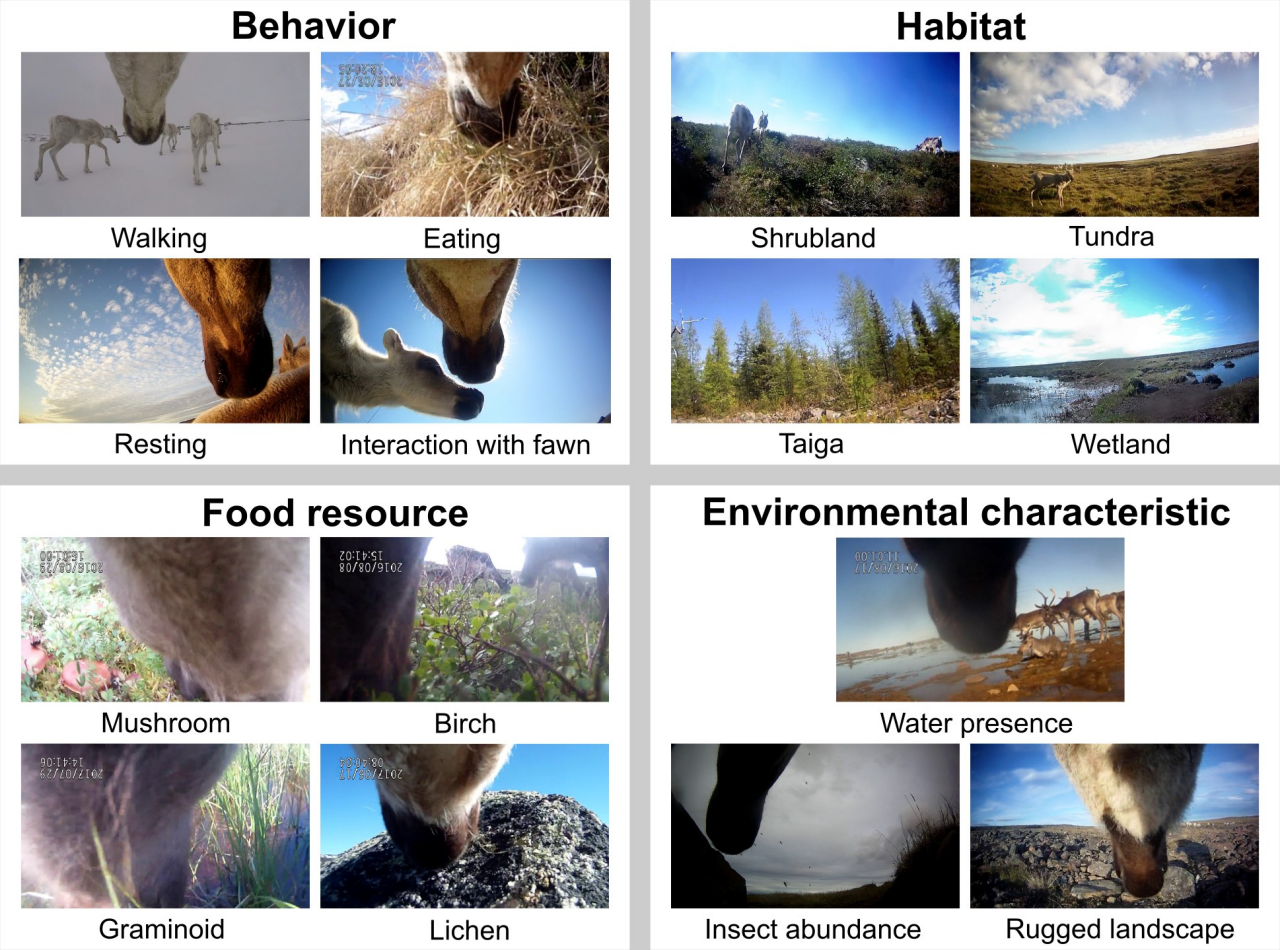
Examples of video stills for behavior, habitat and resource types as well as other environmental conditions. This figure offers a visual example of what we could see in the videos during analysis. Therefore, not all the possible habitat, resource and behavior types are shown. To see complete videos please visit Caribou Ungava (https://www.youtube.com/@caribouungava2647).

We classified the abundance of flying insects such as mosquitoes (Culicidae), black flies (Simuliidae), deer and horse flies (Tabanidae) or botflies (Oestridae) observed on the video. A low abundance was defined as less than 2 insects present around the individual over a 10-sec recording, while the medium and high abundances were defined as 3 to 8 insects and ≥9 insects, respectively. Because insects could only be seen when the caribou had its head up, we used a daily index of insect abundance corresponding to the highest abundance observed for an individual during a given day. We also assessed terrain ruggedness using video footage (Fig 1). We considered terrain as rugged or not by observing if the ground was uneven and if the individual movements were constrained by it (i.e., caribou able or unable to move in a straight line) as was often the case when caribou walked on rocky terrain. If the terrain was smooth but had a steep incline (i.e., around a 30% incline or more), we also included it in the rugged category. Presence or absence of water was determined visually whether it was a lake, a river or a stream (Fig 1). Wetlands were not included in this variable. Finally, at the food resource scale, we classified food resources seen (i.e., consumed or not) into nine groups with similar characteristics, based on family, form, and height (Table 2).

**Table 2:** Description of the resource types used to evaluate the food resource selection of female caribou of the Rivière-aux-Feuilles herd in summer in northern Québec, Canada. Resource types were based on functional groups (i.e., family, form, or height).

Resource Type	Description
Lichens	Includes all growth forms of lichens. Cladoniaceae, Rhizocarpaceae, Parmeliaceae and more
Graminoids	Poaceae, Joncaceae and Cyperaceae
Other shrubs	Mainly Ericaceae but also includes shrubs not identified as birch or willow
Low vegetation	Small plants that are not graminoids and are not higher than about 10 cm, including dwarf species of willow
Other herbaceous	Non shrub plants that are higher than 10 cm. Asteraceae, Rosaseae, Ranunculaceae, Fabaceae, and more
Birches	Betulaceae species, mostly *Betula glandulosa*, *B*. *pumila*, *B*. *minor* and *Alnus alnobetula*
Willows	Salicaceae species in shrub form such as *Salix planifolia*
Mosses	Bryophytes, which includes liverworts, mosses and hornworts
Mushrooms	All families but mainly Boletaceae and Russulaceae

### Meteorological conditions

The meteorological conditions observed in the study area differed among the three years of sampling, which also influenced the timing of vegetation green-up and space use by females. Because the different timing of vegetation green-up could influence the availability of food resources and their selection by caribou, we used green-up date as an indicator for the spring-summer transition and classified each year according to the green-up dates [36]. Approximative green-up dates were determined with Normalized Difference Vegetation Index (NDVI) curves obtained from fine scale NDVI probes located near Deception Bay (62° 09′ 39″ N, 74° 41′ 41″ W), in the northernmost part of the RFH summer range (E. Lemay, unpublished data). Based on the NDVI data, we determined that 2017 was the year with the earliest spring-summer transition (20 June), followed by 2016 (1 July) and 2018 (7 July). We refer to the spring-summer transition simply as summer transition or summer timing for the rest of the text.

### Statistical analyses

At the feeding site scale, we used resource selection probability function (RSPF) [1]. We did modeling using binomial generalized linear mixed models and foraging activity in a video as the response variable (package lme4). Foraging had a value of 1 while the value was 0 for no foraging. Animal identity was included as a random intercept to control for uneven sampling across individuals. To investigate temporal trends in the selection of feeding sites throughout the sampling period, we divided the database into six 2-week periods each year. We arbitrary used a 2-week period considering both the amount of data in a period (statistical power) and the temporal resolution at which we may observe changes in caribou behavior. Variables in the models included the habitat type, terrain ruggedness, water presence, and the daily abundance of insects [5]. We selected the most parsimonious model from a set of candidate models (S2 Table) for each of the biweekly periods based on Akaike’s Information Criterion (AIC) [37]. We assessed model fit of the most parsimonious models using the Receiver Operating Characteristic (ROC) curves [38]. We used the ericaceous tundra as the reference category because its selection ratio was the closest to 1. We based this decision on the fact that, for categorical covariates, the coefficients reflect the ratio of used to available locations for a particular category relative to the ratio of used to available locations for a neutral reference level [39].

At the resources scale, we also used RSPF, but with conditional logistic regression (package “coxme”) to compare resources that were consumed (coded 1) and resources that were not consumed (coded 0), of which there could be many. Resource types were transformed as dummy variables that were coded either as 1 or 0. Therefore, there could be multiple 1 and multiple 0 in each video. We included individual identity as random intercept and video identity as a conditional stratum to pair the observation of consumed and unconsumed food resources. We also divided the database into six 2-week periods for each year. We ran a complete model including all nine food resources (Table 2), but to ensure model convergence we removed food resources that had been used in fewer than 10 videos during a biweekly period (S3 and S5 Tables). We conducted all statistical analyses in R4.0.2 (R Core Team 2020).

## Results

Out of the approximate 200,000 videos recorded by camera collars, we surveyed a total of 65,154 videos, which corresponded to about 181 hours of analyzed footage. We removed an additional 14,191 videos (22%) from the analyses because we could not identify the habitat (e.g., field of view was blocked or too close to the ground). From the remaining 50,963 videos, caribou were observed foraging in 25,869 of them (51%). In the videos where we observed foraging, we mostly saw caribou in tundra and ericaceous tundra in June and July. In 2016, we also observed caribou to frequently forage in wetlands in June and July. In August, caribou mostly foraged in shrublands and tundra with shrubs. The resources most often used in videos were lichens, graminoids, low vegetation, as well as birches in August.

### Feeding site scale

**Fig 2:**
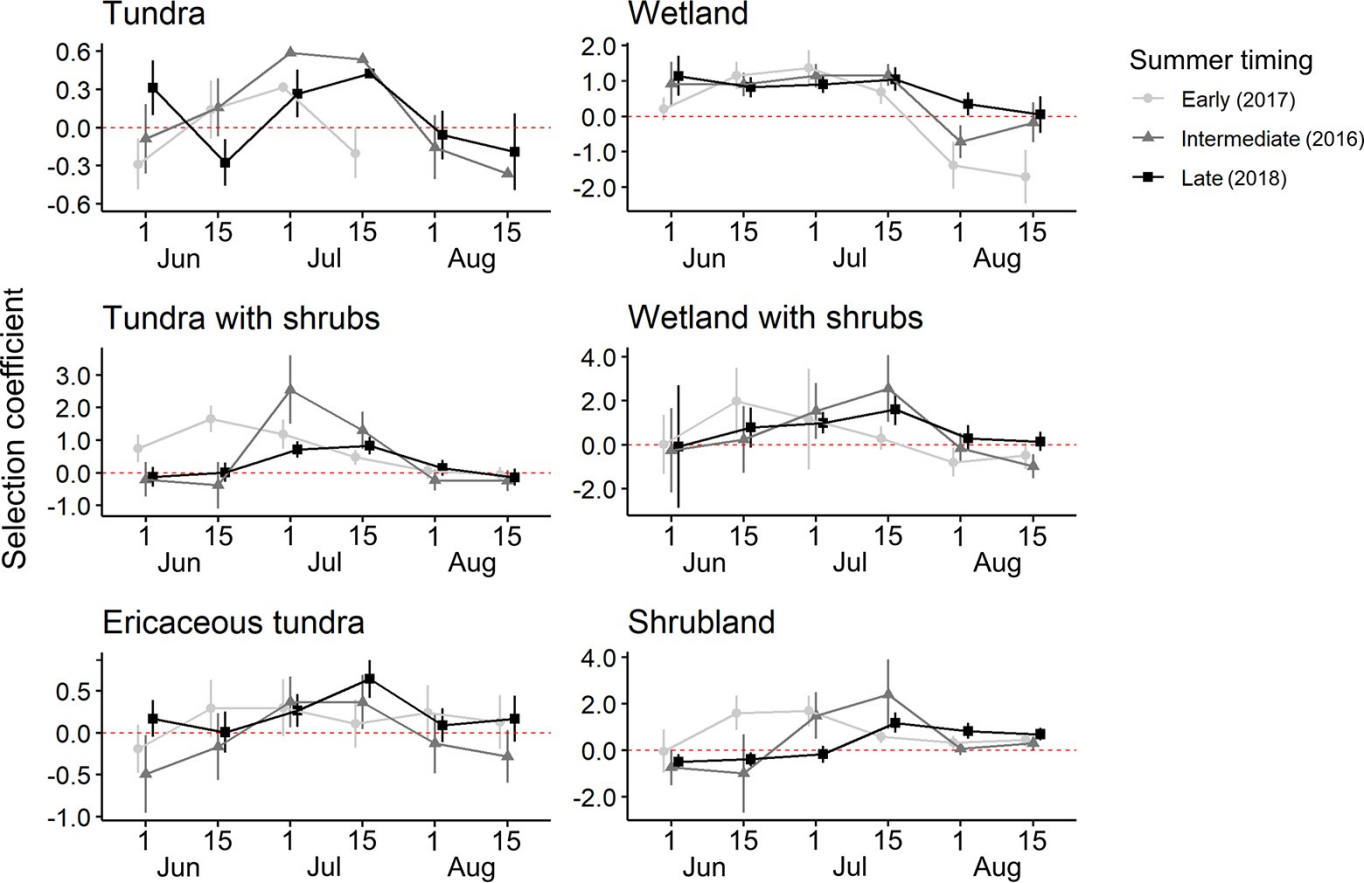
Biweekly selection of feeding sites by female migratory caribou of the Rivière-aux-Feuilles herd during summer for three different summer transitions in northern Québec, Canada. Dates represent the day the two-week period started. Selection coefficients (± 95% CI) obtained with the RSPF were corrected by adding up the associated intercept to the initial coefficient. The red dashed line represents a neutral selection. Summer timing represents the timing of green-up.

**Fig 3:**
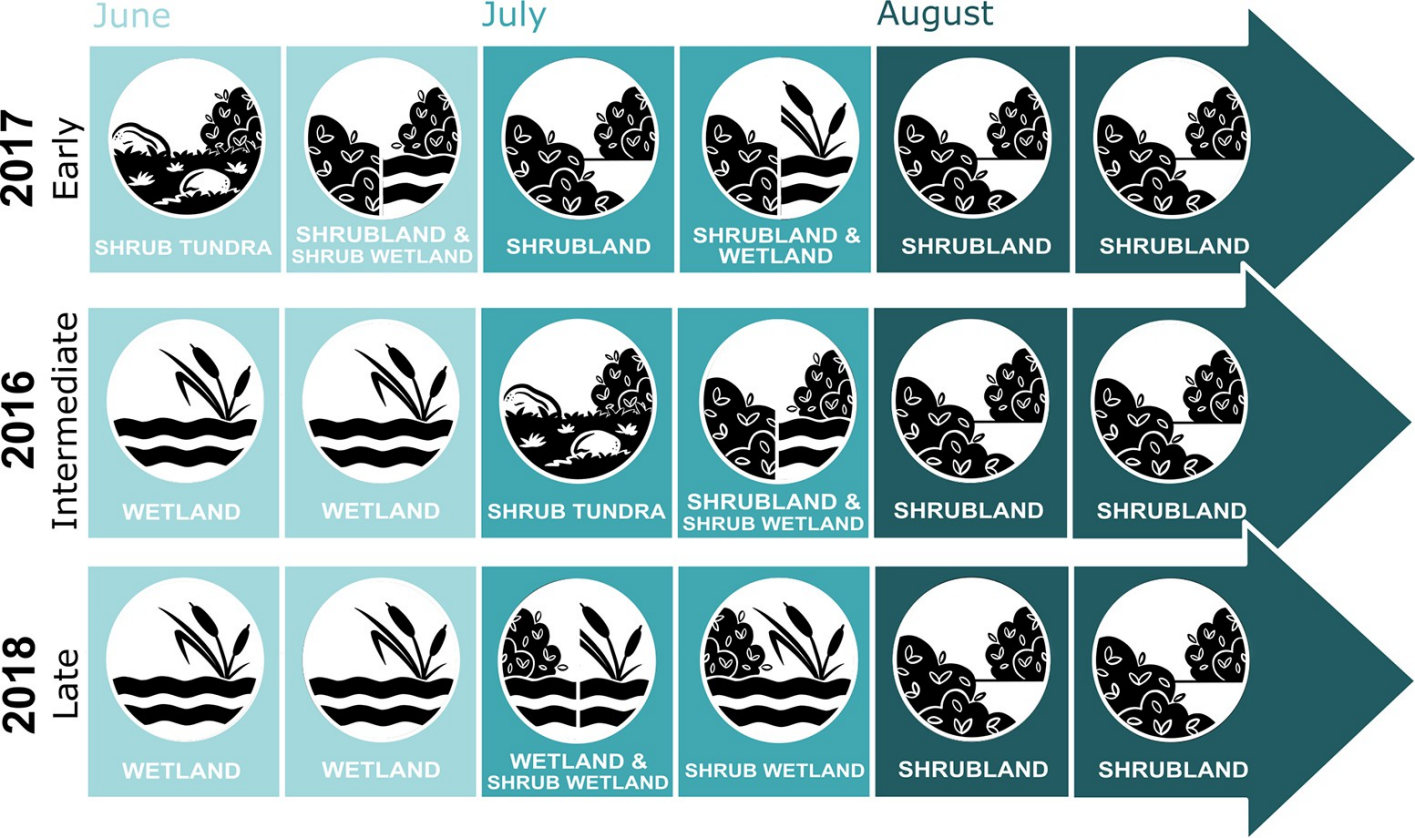
Most selected habitat as feeding site for each two-week period by female migratory caribou of the RFH during summer for three different summer transition periods in northern Québec, Canada.

Of the eight candidate models tested at the feeding site scale, seven were selected as the most parsimonious during at least one period. While the most parsimonious model differed across periods and years, the model including habitat types and terrain ruggedness was consistently selected as the most parsimonious for all three years during the July 1–14 period (S2 Table). The effect or water presence, terrain ruggedness and flying insect abundance on caribou foraging was consistent across periods. Water presence, rugged terrain and medium and high abundance of insects decreased the probability of observing a female caribou foraging (Table 3). Similarly, snowy, and rocky habitat types always had a negative effect on the probability of observing a female caribou foraging (Table 3). Selection for or avoidance of habitat types fluctuated during the summer but almost all habitat types reached a peak of selection followed by a decrease (Fig 2). Generally, wetlands were the most selected habitat type at the start of summer, followed by wetland and tundra with shrubs in midsummer and shrublands at the end of summer (Fig 3). Wetlands were also selected longer during the intermediate (2016) and late (2018) summer transition (Fig 3). We also observed an offset in time for the selection of shrublands the later the summer transition was (Figs 2 and 3).

**Table 3:** Model coefficients and associated 95% confidence intervals (CI) from the most parsimonious model describing feeding site selection by female migratory caribou of the Rivière-aux-Feuilles in northern Québec, Canada.

	Model coefficients (CI 95%)					
Summer timing and variables	June		July		August	
	1	15	1	15	1	15
*Early summer transition (2017)*						
Ericaceous tundra (Intercept)	-0.2(-0.5:0.1)	0.3(0:0.6)	0.3(0:0.6)	0.1(-0.2:0.4)	0.2(-0.1:0.6)	0.1(-0.2:0.4)
Tundra with shrubs	**0.9(0.5:1.4)**	**1.4(1:1.8)**	**0.9(0.4:1.3)**	**0.4(0.1:0.6)**	-0.2(-0.4:0.1)	-0.2(-0.4:0.1)
Tundra	-0.1(-0.3:0.1)	-0.2(-0.4:0.1)	0(-0.3:0.3)	**-0.3(-0.5:-0.1)**	**-1.3(-1.6:-1)**	**-1.4(-1.7:-1.1)**
Wetland with shrubs	0.2(-1.1:1.5)	**1.7(0.2:3.2)**	0.9(-1.4:3.2)	0.2(-0.3:0.7)	**-1(-1.7:-0.4)**	-0.6(-1.2:0)
Wetland	**0.4(0.1:0.7)**	**0.9(0.5:1.2)**	**1.1(0.6:1.6)**	**0.6(0.2:0.9)**	**-1.6(-2.3:-1)**	**-1.8(-2.6:-1.1)**
Shrubland	0.1(-0.8:1.1)	**1.3(0.6:2)**	**1.4(0.7:2)**	**0.5(0.2:0.8)**	0.1(-0.2:0.4)	0.3(0:0.6)
Rocky ground	0.1(-0.8:1)	-1.1(-2.2:0)	**-1.9(-3.2:-0.6)**	**-2.1(-2.8:-1.4)**	-15.5(-86.4:55.5)	**-3.8(-5.8:-1.8)**
Snowy ground	**-2.4(-3.2:-1.6)**	NA	NA	NA	NA	NA
Taiga	NA	NA	NA	NA	-1.5(-3.1:0.2)	**-0.7(-1.3:-0.1)**
Water presence	NA	NA	NA	NA	NA	NA
Ruggedness	**-1(-1.6:-0.5)**	NA	**-1(-1.3:-0.6)**	**-0.7(-1.0:-0.3)**	**-0.8(-1.5:-0.07)**	-14.8(-70.2:40.6)
Insect abundance						
low	NA	-0.2(-0.4:0)	NA	**-0.3(-0.4:-0.09)**	-0.1(-0.3:0.09)	**-0.3(-0.5:-0.09)**
medium	NA	**-0.4(-0.7:-0.1)**	NA	**-0.3(-0.5:-0.03)**	**-0.4(-0.6:-0.2)**	**-0.5(-0.7:-0.2)**
high	NA	-0.1(-0.6:0.4)	NA	**-0.5(-0.8:-0.3)**	**-0.6(-0.8:-0.3)**	**-0.5(-0.7:-0.3)**
*Intermediate summer transition (2016)*						
Tundra with shrubs	0.3(-0.2:0.8)	-0.2(-0.9:0.5)	**2.2(1.1:3.2)**	**0.9(0.4:1.5)**	-0.1(-0.4:0.2)	0(-0.3:0.4)
Tundra	**0.4(0.1:0.7)**	**0.3(0.1:0.6)**	0.2(-0.1:0.5)	0.2(-0.1:0.4)	0(-0.3:0.2)	-0.1(-0.5:0.3)
Wetland with shrubs	0.2(-1.7:2.1)	0.4(-1.1:1.9)	1.2(-0.1:2.4)	**2.2(0.7:3.7)**	0(-0.7:0.7)	**-0.7(-1.3:-0.2)**
Wetland	**1.4(0.8:2)**	**1.1(0.7:1.4)**	**0.8(0.4:1.1)**	**0.8(0.5:1.1)**	**-0.6(-1.1:-0.1)**	0.1(-0.5:0.7)
Shrubland	-0.2(-1:0.5)	-0.8(-2.5:0.8)	**1.1(0.1:2.1)**	**2(0.5:3.5)**	0.2(-0.1:0.5)	**0.6(0.3:0.9)**
Rocky ground	**-1.8(-2.4:-1.2)**	**-1.4(-1.9:-0.9)**	**-2.8(-4.3:-1.3)**	**-3.7(-4.8:-2.5)**	**-2.5(-3.1:-1.8)**	**-2.8(-4.3:-1.4)**
Snowy ground	**-1.5(-1.9:-1)**	NA	NA	NA	NA	NA
Taiga	NA	NA	NA	NA	0.2(-2.2:2.7)	0(-0.5:0.4)
Water presence	-1.3(-2.7:0.05)	NA	NA	**-0.8(-1.5:-0.1)**	NA	-1.4(-1.8:0.3)
Ruggedness	NA	**-0.5(-0.8:-0.2)**	NA	**-0.9(-1.2:-0.6)**	**-0.2(-1.3:-0.6)**	**-4.2(-1.2:-0.4)**
Insect abundance						
low	NA	NA	NA	-0.06(-0.3:0.1)	NA	-0.04(-0.24:0.16)
medium	NA	NA	NA	0.1(-0.3:0.5)	NA	**-0.3(-0.6:-0.1)**
high	NA	NA	NA	-**0.5(-0.9:-0.1)**	NA	**-0.4(-0.7:-0.01)**
*Late summer transition (2018)*						
Ericaceous tundra (Intercept)	0.2(0:0.4)	0(-0.2:0.3)	**0.3(0.1:0.5)**	**0.6(0.4:0.9)**	0.1(-0.1:0.3)	**0.2(-0.1:0.4)**
Tundra with shrubs	**-0.3(-0.6:0)**	0(-0.3:0.3)	**0.4(0.2:0.7)**	0.2(-0.1:0.5)	0.1(-0.2:0.3)	-0.3(-0.6:0)
Tundra	0.1(-0.1:0.4)	**-0.3(-0.5:-0.1)**	**0(-0.2:0.2)**	**-0.2(-0.4:0)**	-0.1(-0.3:0)	**-0.4(-0.7:-0.1)**
Wetland with shrubs	-0.3(-3.1:2.5)	0.8(-0.1:1.7)	**0.7(0.2:1.2)**	**1(0.3:1.7)**	0.2(-0.4:0.8)	0(-0.4:0.4)
Wetland	**1(0.4:1.5)**	**0.8(0.5:1.1)**	**0.6(0.4:0.9)**	**0.4(0.1:0.7)**	0.3(-0.1:0.6)	-0.1(-0.6:0.4)
Shrubland	**-0.7(-1:-0.3)**	**-0.4(-0.7:-0.1)**	**-0.4(-0.8:-0.1)**	**0.5(0.1:1)**	**0.7(0.4:1.1)**	**0.5(0.3:0.8)**
Rocky ground	0.7(-0.5:1.9)	**0.8(0.1:1.5)**	0.3(-0.5:1.1)	**-1.1(-1.8:-0.4)**	**-1.8(-2.3:-1.2)**	**-1.4(-2.4:-0.4)**
Snowy ground	**-2.2(-2.5:-1.9)**	NA	NA	NA	NA	NA
Taiga	NA	NA	NA	NA	NA	0.1(-0.4:0.5)
Water presence	NA	NA	NA	NA	NA	**-1.2(-2:-0.4)**
Ruggedness	NA	NA	**-2.2(-3.8:-0.7)**	**-1.5(-2.4:-0.5)**	NA	NA
Insect abundance						
low	NA	NA	NA	**-0.2(-0.3:-0.02)**	0.09(-0.08:0.3)	**-0.3(-0.4:-0.1)**
medium	NA	NA	NA	**-0.3(-0.6:-0.08)**	**-0.4(-0.7:-0.08)**	**-0.3(-0.6:-0.02)**
high	NA	NA	NA	**-0.8(-1.1:-0.5)**	-0.3(-0.6:0.06)	-0.3(-0.6:0.02)

Each date represents the start of a two-week period. Summer transition represents the timing of green-up.

### Food item scale

**Fig 4:**
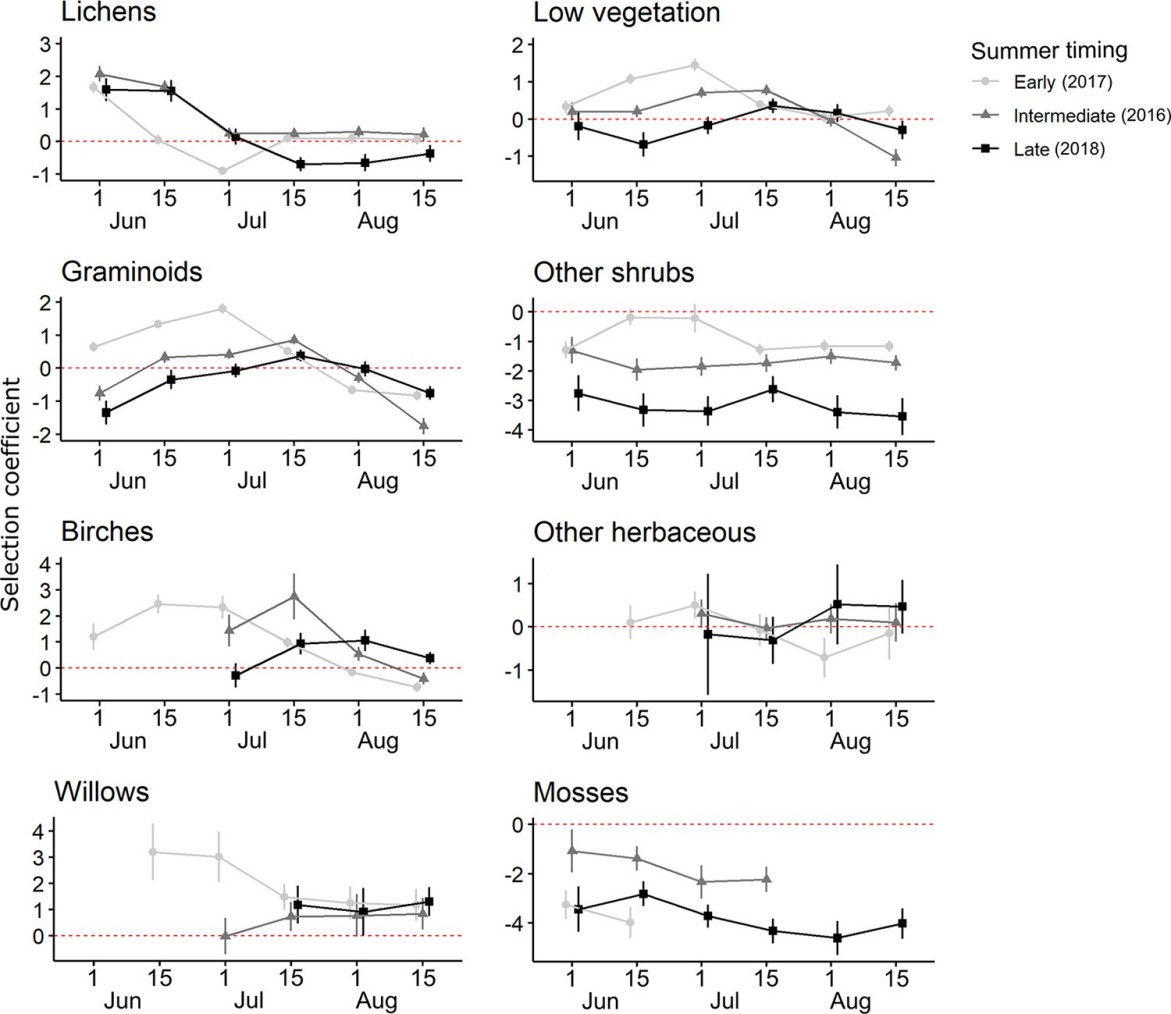
Biweekly selection of food resources by female migratory caribou of the RFH during summer for three different summer transitions in northern Québec, Canada. Dates represent the day the two-week period started. Selection coefficients (± 95% CI) were obtained with cox models. The red dashed line represents neutral selection. Summer timing represents the timing of green-up.

**Fig 5:**
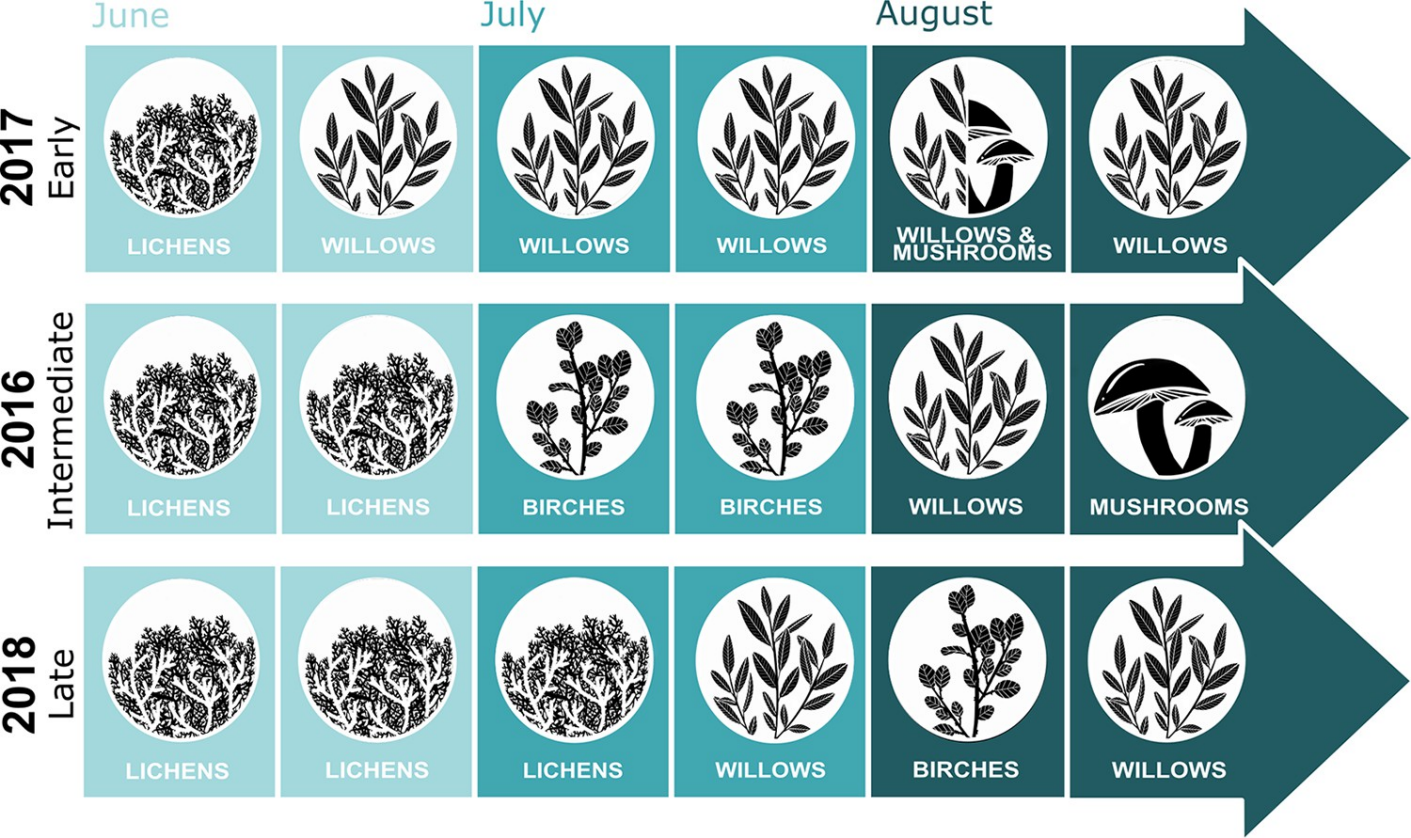
Food resources with the strongest selection by female migratory caribou of the RFH for each two-week period during summer for three different summer transitions in northern Québec, Canada.

At the food resources scale, mosses and other shrubs (i.e., shrubs other than birches or willows) were avoided throughout all periods (Table 4; Fig 4). Selection of most food resources fluctuated during summer. Selection of some resources such as lichens, low vegetation, graminoids and birches reached a peak followed by a decrease (Fig 4). Selection trends were similar across years for most resources, except that the selection often presented an offset in time, namely that selection for resources occurred earlier during the summer when the summer transition was earlier (2017; Fig 4). Trends in resource selection tended to be more similar between the intermediate (2016) and late (2018) transition summers (Fig 4). Graminoids, low vegetation and willows were more strongly selected in 2017 compared to 2016 and 2018 (Table 4; Figs 4 and 5). Birches were strongly selected during late-June and early-July in 2017 and during July in 2016 (Fig 5). For all years, lichens were selected in early-June, but the selection decreased throughout the summer, particularly when the summer transition was early (Fig 5). Resources removed from models for convergence were either strongly avoided by caribou or not present/available enough in the landscape (S3 and S5 Tables). For mosses, they were readily available to caribou and strongly avoided in each period when we were able to put them in our models. On the other hand, mushrooms were unavailable except in the two last periods of August (depending on the year). Similarly, willows and other herbaceous were not available in early summer.

**Table 4:** Model coefficients and associated 95% confidence intervals (CI) from the complete model describing resource selection by female migratory caribou of the RFH in northern Québec, Canada.

		Model coefficients (CI 95%)				
Summer timing and variables	June		July		August	
	1	15	1	15	1	15
*Early summer transition (2017)*						
Lichens	**1.7(1.5:1.8)**	0.1(-0.1:0.2)	**-0.9(-1.1:-0.7)**	0.1(0:0.2)	0.1(0:0.2)	0.1(-0.1:0.2)
Graminoids	**0.6(0.5:0.8)**	**1.3(1.2:1.5)**	**1.8(1.7:2)**	**0.5(0.4:0.6)**	**-0.7(-0.8:-0.5)**	**-0.8(-1:-0.7)**
Other shrubs	**-1.3(-1.6:-1)**	-0.2(-0.5:0.1)	-0.2(-0.7:0.3)	**-1.3(-1.5:-1.1)**	**-1.1(-1.3:-0.9)**	**-1.2(-1.4:-1)**
Low vegetation	**0.3(0.2:0.5)**	**1.1(0.9:1.2)**	**1.5(1.3:1.6)**	**0.4(0.3:0.5)**	0(-0.1:0.2)	**0.2(0.1:0.4)**
Mosses	**-3.3(-3.8:-2.7)**	**-4(-4.6:-3.3)**	NA	NA	NA	NA
Other herbaceous	NA	0.1(-0.3:0.5)	**0.5(0.2:0.8)**	-0.1(-0.4:0.3)	**-0.7(-1.2:-0.3)**	-0.1(-0.8:0.5)
Birches	**1.2(0.7:1.7)**	**2.5(2.1:2.8)**	**2.3(1.9:2.8)**	**1(0.8:1.2)**	-0.1(-0.3:0)	**-0.7(-0.9:-0.6)**
Willows	NA	**3.2(2.1:4.3)**	**3(2:4)**	**1.5(1:2)**	**1.2(0.6:1.9)**	**1.2(0.6:1.8)**
Mushrooms	NA	NA	NA	NA	**1.3(0.6:1.9)**	**0.7(0.1:1.4)**
*Intermediate summer transition (2016)*						
Lichens	**2.1(1.8:2.3)**	**1.7(1.5:1.9)**	**0.2(0.1:0.4)**	**0.2(0.1:0.4)**	**0.3(0.1:0.5)**	0.2(0:0.4)
Graminoids	**-0.8(-1:-0.5)**	**0.3(0.2:0.5)**	**0.4(0.3:0.6)**	**0.9(0.7:1)**	**-0.3(-0.4:-0.1)**	**-1.8(-2:-1.5)**
Other shrubs	**-1.3(-1.8:-0.9)**	**-2(-2.3:-1.6)**	**-1.8(-2.2:-1.5)**	**-1.7(-2:-1.4)**	**-1.5(-1.8:-1.2)**	**-1.7(-2:-1.5)**
Low vegetation	**0.2(0:0.4)**	0.2(0:0.4)	**0.7(0.6:0.9)**	**0.8(0.6:0.9)**	0(-0.2:0.1)	**-1(-1.3:-0.8)**
Mosses	**-1.1(-1.9:-0.2)**	**-1.4(-1.9:-0.9)**	**-2.3(-3:-1.7)**	**-2.2(-2.7:-1.7)**	NA	NA
Other herbaceous	NA	NA	0.3(0:0.6)	0(-0.3:0.2)	0.2(-0.2:0.5)	0.1(-0.3:0.5)
Birches	NA	NA	**1.4(0.8:2.1)**	**2.7(1.9:3.6)**	**0.5(0.3:0.8)**	**-0.4(-0.6:-0.2)**
Willows	NA	NA	0(-0.7:0.7)	**0.7(0.2:1.3)**	**0.8(0:1.6)**	**0.8(0.2:1.5)**
Mushrooms	NA	NA	NA	NA	NA	**1.1(0.1:2.2)**
*Late summer transition (2018)*						
Lichens	**1.6(1.2:1.9)**	**1.6(1.2:1.9)**	0.1(-0.1:0.4)	**-0.7(-0.9:-0.5)**	**-0.7(-0.9:-0.4)**	**-0.4(-0.6:-0.1)**
Graminoids	**-1.3(-1.7:-1)**	-0.3(-0.6:0)	-0.1(-0.3:0.1)	**0.4(0.2:0.6)**	0(-0.3:0.2)	**-0.8(-1:-0.5)**
Other shrubs	**-2.8(-3.4:-2.2)**	**-3.3(-3.9:-2.8)**	**-3.4(-3.9:-2.9)**	**-2.6(-3.1:-2.2)**	**-3.4(-3.9:-2.8)**	**-3.5(-4.2:-2.9)**
Low vegetation	-0.2(-0.6:0.2)	**-0.7(-1:-0.4)**	-0.2(-0.4:0.1)	**0.4(0.2:0.5)**	0.2(-0.1:0.4)	-0.3(-0.5:0)
Mosses	**-3.4(-4.4:-2.5)**	**-2.8(-3.3:-2.3)**	**-3.7(-4.2:-3.3)**	**-4.3(-4.8:-3.8)**	**-4.6(-5.3:-3.9)**	**-4(-4.6:-3.4)**
Other herbaceous	NA	NA	-0.2(-1.6:1.2)	-0.3(-0.9:0.2)	0.5(-0.4:1.4)	0.5(-0.2:1.1)
Birches	NA	NA	-0.3(-0.7:0.2)	**0.9(0.5:1.3)**	**1.1(0.7:1.5)**	**0.4(0.1:0.6)**
Willows	NA	NA	NA	**1.2(0.5:1.9)**	0.9(0:1.8)	**1.3(0.7:1.9)**
Mushrooms	NA	NA	NA	NA	NA	-0.1(-2.8:2.6)

Each date represents the start of a two-week period. Summer transition represents the timing of green-up.

## Discussion

We investigated female migratory caribou habitat selection during summer at the third and fourth orders of selection (sensu Johnson [2]) using video cameras mounted on GPS collars. Generally, female caribou selected wetlands, shrublands and tundra to feed, and the food items that were mostly selected were lichens, birches, willows, and mushrooms. By using a new technology, we showed as hypothesized how caribou adjust their feeding ecology depending on resource availability and other environmental variables, such as terrain ruggedness and flying insect harassment.

At the feeding site scale, our results revealed that wetlands were selected for a longer period when summer arrived later. Wetlands could be a key habitat at the start of the summer transition for female caribou, as they can offer one of the first green resources of the year, i.e., freshly grown graminoids [23]. After a long winter of eating mainly lichens, access to nutritious resources could help females face the costs of the last gestation phase, lactation, and migration [40–42]. Trends in the selection of feeding sites were similar from one year to another but showed an offset depending on the time when the summer transition occurred. Resource selection seems to concur with the vegetation phenology, consistent with other studies [43, 44], but it is difficult to conclude on the robustness of this correlation because we only had 3 years of data. This offset, however, disappeared in August, when shrublands were the most selected habitat regardless of the timing of the summer transition. With the window of plant availability being key to face subsequent survival needs [45], a shorter window and later phenology could also have consequences for calf viability, since it is correlated with female condition at parturition.

As predicted, we detected an effect of daily flying insect abundance on the feeding behavior of female caribou. Indeed, even a low index of insect harassment had a negative effect on the probability of observing a female caribou feeding. When subjected to insect harassment, caribou were shaking their heads, staying immobile with their heads down, or walking or running away instead of feeding. Such behaviors were also observed in other studies on caribou/reindeer [14, 46, 47]. Indeed, Russel, Martell and Nixon [46] found a decrease in the foraging budgets of caribou exposed to insect harassment. Warming temperatures and climate change could increase the abundance of insects and therefore negatively impact foraging behavior. Although each species of insects has a different life cycle, it was found that survival of mosquito larvae was higher at temperatures ranging from 20 to 30 ˚C and decreased at lower or higher temperatures [48]. The period during which peak temperatures occur in summer also influences mosquito abundance [49]. During a late summer, when temperatures peak in August, the peak in insect abundance is higher than when peak temperatures occur in July [49]. Since August is usually the last month to benefit from summer food resources, increased harassment in August could mean less time spent foraging, with negative consequences on caribou. Terrain ruggedness also had a negative effect on foraging behavior. Rugged terrains were observed mostly in habitats where resources were less available and although we sometimes saw caribou eat lichens on rocks, they mostly passed through rugged habitats without foraging, explaining the negative effect observed.

Our results showed that lichens represented a large part of the consumed resources and were strongly selected, but only when other resources were less available (i.e., start of June, from 41 to 63% of used resources; S3 Table) or when the quality of other resources started to decrease in August (i.e., end of August 25 to 38%; S3 Table). Lichens are poor in minerals and proteins [50], therefore, when more nutritious resources were present in a patch, caribou avoided lichens and used other resources such as leaves of deciduous shrubs, especially when new leaves came out. Lichens were sometimes selected or eaten when caribou were eating low vegetation. These results support the claim that caribou is not an obligate lichen forager in summer, but they rather use and select lichens when necessary, such as when other resources are scarce [25, 27]. Mosses are not a good quality resource for caribou. They have a high fiber content and low protein concentrations [51, 52], and they were avoided by caribou throughout summer. Mosses were incidentally consumed with lichens or low vegetation, as seen in other studies [27, 36, 51]. Other shrubs, which mostly consisted of ericaceous shrubs, were also avoided throughout summer. Most ericaceous shrubs produce secondary compounds making them less palatable for caribou [53]. Birches also produce secondary compounds like phenols and tannins which are more abundant later in the season, reducing their digestibility [54–56], which may explain why caribou avoided birches at the end of the summer of 2016 and 2017. In 2018, during a late summer transition year, caribou selected birches until the end of the summer, possibly because birches had not reached the same growth stage as during the two other summers and had produced fewer secondary compounds. Although birches were sometimes avoided at the end of summer, they represented 11 to 31% of the observed used resources across years (S3 Table). Species of willows, which generally contain fewer phenols, tannins and more proteins than birches [56], were selected but represented only 1 to 5% of all resources used (S3 Table) because they were not as available as birches. At the end of summer, female caribou showed a strong selection for mushrooms in 2016 and 2017. Because plant phenology was delayed in 2018, we might have seen a similar selection for mushrooms if our data collection had been extended. Although scarce, mushrooms are a good source of proteins and vitamins and could represent a good boost in nutrients when other resources start to become less nutritious or scarce [50, 57, 58]. We often observed females eating mushrooms before consuming any other resources in a feeding site. Selection of the most nutritious resources in summer may allow females to build enough fat reserves to increase the viability of their next offspring [59, 60].

Camera collars were a great way to observe and quantify animal behavior in the wild, but they also have limitations. For instance, we could not count the number of bites taken because of the angle of the camera and we could not identify resources at the species level in many cases. We would recommend using cameras with a definition of 1080p or higher to identify resources consumed at the species level. Also, while we are confident that we have covered most caribou feeding behavior during summer, we analyzed footage from 05:00 to 20:00 and may have therefore missed different foraging patterns occurring at night. Nevertheless, our study revealed a much more precise portrait of the summer foraging and selection patterns of female migratory caribou than could be obtained using other approaches. Along with other studies [27, 36], we confirmed that female caribou are selective in their feeding sites and resource selection. This is especially true when more resources are available in a patch and where females can afford to be selective and choose the most nutritious resource available.

## Supporting information

S1 File French version of the abstract. (DOCX)

S1 Table Fixed effect structure of the most parsimonious logistic regression models used to describe feeding site selection by female migratory caribou of the RFH at each biweekly period in northern Québec, Canada. Variables include the habitat type (HAB), terrain ruggedness (RUGG), water presence (WPRES), and the daily abundance of insects (INS). We also provide the area under the curve (AUC) of the Receiver Operating Characteristic (ROC) curves. Models also include animal identity as a random intercept. Summer transition represents the timing of green-up.
(DOCX)

S2 Table Candidate models tested to determine the effects of habitat types, water presence, terrain ruggedness, and insect abundance on the probability that female caribou were using a site for feeding. For each model, we provide the Log Likelihood (LL), delta AIC (Δ AIC), and model weight (wi).
(DOCX)

S3 Table Percentages of videos where a food resource type was observed as consumed by female migratory caribou of the RFH, in northern Québec, Canada. Each date represents the start of a two-week period. Percentages were calculated as the number of videos where a resource was consumed at least once divided by the total number of videos for that period. Multiple resource types could be consumed in one video.
(DOCX)

S4 Table Percentages of videos where a habitat type was used as a feeding site at least once by female migratory caribou of the RFH, in northern Québec, Canada. Each date represents the start of a two-week period. Percentages were calculated with the number of videos where a habitat was seen as used and the total number of videos for that period.
(DOCX)

S5 Table Percentages of videos where a food resource type was observed as present and unconsumed. Each date represents the start of a two-week period. Percentages were calculated with the number of videos where a resource was present and unconsumed at least once and the total number of videos for that period. Multiple resource types could be present in one video.
(DOCX)

S6 Table Set of examples videos recorded by camera collars on migratory caribou of the Rivière-aux-Feuilles herd in summer in northern Quebec, Canada. For description of each habitat, resource types and other variables, please see main article. We considered a site used when an individual foraged at least once during a video. On the contrary, if an individual was exhibiting any other behavior (e.g., resting, walking, vigilance) without foraging, we considered this site to be unused.
(DOCX)

S1 Fig Population size (x1000) of the Rivière-aux-Feuilles (RFH) and the Rivière-George (RGH) migratory caribou herds, in northern Québec, Canada. Points without a confidence interval represents a minimal count and had no error associated to it [20].
(TIF)
