# Supplementary material for: Selection of summer feeding sites and food resources by female migratory caribou (Rangifer tarandus) determined using camera collars
Source: PLoS One. 2023 Nov 29;18(11):e0294846. doi: 10.1371/journal.pone.0294846 (PMC10686509; doi:10.1371/journal.pone.0294846)
Supplement: S1 File — (DOCX) [file pone.0294846.s001.docx]

**S6 Abstract**

Le caribou migrateur (*Rangifer tarandus*) est une espèce clé sur le plan socio-économique et culturel pour les communautés nordiques du Québec, et ses populations connaissent un fort déclin. Les femelles caribous migrateurs dépendent de la disponibilité des ressources de l'habitat estival pour répondre aux besoins associés à la lactation et à l'accumulation de réserves de graisse pour survivre lorsque les ressources sont moins abondantes. En raison des larges échelles auxquelles les données sur l'habitat et les ressources sont habituellement disponibles, il existe peu d'informations sur la façon dont les femelles caribous migrateurs sélectionnent l'habitat et les ressources à de fines échelles en milieu naturel. Pour documenter la sélection des sites d'alimentation estivaux, nous avons équipé 60 caribous femelles de colliers caméras de 2016 à 2018. Nous avons collecté un total de 65 150 vidéos de 10 secondes entre le 1^er^ juin et le 1er septembre pour trois années présentant une phénologie printanière contrastée. Nous avons comparé les sites utilisés pour l’alimentation aux sites utilisés pour toute autre activité (3^e^ échelle de Johnson) et à l'échelle de l'article alimentaire (4^e^ échelle de Johnson) en utilisant des fonctions de sélection des ressources. Les zones humides ont été fortement sélectionnées comme sites d'alimentation en juin et juillet alors qu'elles ont été évitées en août. Les zones arbustives ont été principalement choisies en juillet et août. À l'échelle des ressources, le lichen, le bouleau, le saule et les champignons étaient les ressources les plus fortement sélectionnées. Nos résultats fournissent des informations précises et nouvelles sur la sélection de l'habitat sur les sites d'alimentation et les ressources alimentaires sélectionnées par les femelles caribous dans la nature. Ces informations aideront à comprendre les habitudes d'alimentation et le comportement de sélection de l'habitat des caribous migrateurs femelles et contribueront à la gestion et à la conservation de leurs populations en déclin.

**Mots clé:** caribou migrateur, sélection d’habitat à fine échelle, colliers caméra, fonction de sélection des ressources, site d’alimentation
