# Supplementary material for: Selection of summer feeding sites and food resources by female migratory caribou (Rangifer tarandus) determined using camera collars
Source: PLoS One. 2023 Nov 29;18(11):e0294846. doi: 10.1371/journal.pone.0294846 (PMC10686509; doi:10.1371/journal.pone.0294846)
Supplement: S1 Table — Variables include the habitat type (HAB), terrain ruggedness (RUGG), water presence (WPRES), and the daily abundance of insects (INS). We also provide the area under the curve (AUC) of the Receiver Operating Characteristic (ROC) curves. Models also include animal identity as a random intercept. Summer transition represents the timing of green-up. (DOCX) [file pone.0294846.s002.docx]

**S1 Table** Fixed effect structure of the most parsimonious logistic regression model used to describe the feeding site selection of female migratory caribou at each biweekly period. Variables include the habitat type (HAB), terrain ruggedness (RUGG), water presence (WPRES, and the daily abundance of insects (INS). We also provide the area under the curve (AUC) of the Receiver Operating Characteristic (ROC) curves. Models also include animal identity as a random intercept.

|  | **Summer transition type** | | | | |  |
| --- | --- | --- | --- | --- | --- | --- |
|  | **Early (2017)** | | **Intermediate (2016)** | | **Late (2018)** | |
| **Biweekly period** | **Model** | **AUC** | **Model** | **AUC** | **Model** | **AUC** |
| June 1-14 | HAB+RUGG | 0.67 | HAB+WPRES | 0.74 | HAB | 0.70 |
| June 15-30 | HAB+INS | 0.66 | HAB+RUGG | 0.69 | HAB | 0.64 |
| July 1-14 | HAB+RUGG | 0.64 | HAB+RUGG | 0.67 | HAB+RUGG | 0.62 |
| July 15-31 | HAB+RUGG+INS | 0.67 | HAB+RUGG+WPRES+INS | 0.70 | HAB+RUGG+INS | 0.62 |
| August 1-14 | HAB+RUGG+INS | 0.71 | HAB+RUGG | 0.70 | HAB+INS | 0.63 |
| August 15-31 | HAB+RUGG+WPRES+INS | 0.71 | HAB+RUGG+WPRES+INS | 0.65 | HAB+INS+WPRES | 0.64 |
