# Supplementary material for: Selection of summer feeding sites and food resources by female migratory caribou (Rangifer tarandus) determined using camera collars
Source: PLoS One. 2023 Nov 29;18(11):e0294846. doi: 10.1371/journal.pone.0294846 (PMC10686509; doi:10.1371/journal.pone.0294846)
Supplement: S2 Table — For each model, we provide the Log Likelihood (LL), delta AIC (Δ AIC), and model weight (wi). (DOCX) [file pone.0294846.s003.docx]

| **Period** | **Model** | | **Early summer (2017)** | | | **Intermediate summer (2016)** | | | **Late summer (2018)** | | |
| --- | --- | --- | --- | --- | --- | --- | --- | --- | --- | --- | --- |
|  |  |  | **LL** | **∆ AIC** | ***w_i_*** | **LL** | **∆ AIC** | ***w_i_*** | **LL** | **∆ AIC** | ***w_i_*** |
| June 1-14 | Habitats | | -1649.2 | 12.4 | 0.0 | -974.52 | 1.7 | 0.2 | **-1819.0** | **0.0** | **0.4** |
|  | Habitats + Water presence | | -1649.0 | 13.9 | 0.0 | **-972.67** | **0.0** | **0.3** | -1818.9 | 1.9 | 0.1 |
|  | Habitats + Ruggedness | | **-1642.0** | **0.0** | **0.5** | -974.45 | 3.6 | 0.1 | -1818.7 | 1.4 | 0.2 |
|  | Habitats + Insect | | -1647.5 | 15.0 | 0.0 | -974.23 | 3.1 | 0.1 | -1818.2 | 2.4 | 0.1 |
|  | Habitats + Water presence + Ruggedness | | -1641.8 | 1.6 | 0.2 | -972.64 | 2.0 | 0.1 | -1818.6 | 3.3 | 0.1 |
|  | Habitats + Water presence + Insect abundance | | -1647.2 | 16.5 | 0.0 | -972.39 | 1.5 | 0.2 | -1818.1 | 4.3 | 0.0 |
|  | Habitats + Ruggedness + Insect abundance | | -1640.3 | 2.6 | 0.2 | -974.16 | 5.0 | 0.0 | -1817.9 | 3.8 | 0.1 |
|  | Global |  | -1640.1 | 4.2 | 0.1 | -972.36 | 3.4 | 0.1 | -1817.8 | 5.7 | 0.0 |
|  | Null |  | -1699.0 | 97.8 | 0.0 | -1069.3 | 177.2 | 0.0 | -2015.1 | 378.3 | 0.0 |
| June 15-30 | Habitats | | -1912.9 | 3.1 | 0.1 | -1226.2 | 6.6 | 0.0 | **-2183.7** | **0.0** | **0.3** |
|  | Habitats + Water presence | | -1912.9 | 5.1 | 0.0 | -1226 | 8.2 | 0.0 | -2183.4 | 1.4 | 0.2 |
|  | Habitats + Ruggedness | | -1912.4 | 4.1 | 0.1 | **-1221.9** | **0.0** | **0.4** | -2183.7 | 1.9 | 0.1 |
|  | Habitats + Insect abundance | | **-1908.3** | **0.0** | **0.4** | -1224.1 | 6.5 | 0.0 | -2183.7 | 1.9 | 0.1 |
|  | Habitats + Water presence + Ruggedness | | -1912.4 | 6.0 | 0.0 | -1221.7 | 1.6 | 0.2 | -2183.3 | 3.2 | 0.1 |
|  | Habitats + Water presence + Insect abundance | | -1908.3 | 2.0 | 0.1 | -1223.9 | 8.0 | 0.0 | -2183.4 | 3.3 | 0.1 |
|  | Habitats + Ruggedness + Insect abundance | | -1907.7 | 0.7 | 0.3 | -1220.0 | 0.3 | 0.3 | -2183.6 | 3.8 | 0.1 |
|  | Global |  | -1907.7 | 2.7 | 0.1 | -1219.8 | 1.8 | 0.1 | -2183.3 | 5.2 | 0.0 |
|  | Null |  | -1974.5 | 114.2 | 0.0 | -1281.3 | 104.8 | 0.0 | -2220.5 | 61.5 | 0.0 |
| July  1-14 | Habitats | | -1891.0 | 24.3 | 0.0 | -1040.3 | 13.3 | 0.0 | -2022.1 | 8.8 | 0.0 |
|  | Habitats + Water presence | | -1890.5 | 25.3 | 0.0 | -1040.0 | 14.7 | 0.0 | -2021.3 | 9.2 | 0.0 |
|  | Habitats + Ruggedness | | **-1877.8** | **0.0** | **0.5** | **-1032.6** | **0.0** | **0.6** | **-2016.7** | **0.0** | **0.4** |
|  | Habitats + Insect abundance | | -1888.5 | 25.2 | 0.0 | -1038.7 | 16.1 | 0.0 | -2020.2 | 11.1 | 0.0 |
|  | Habitats + Water presence + Ruggedness | | -1877.6 | 1.5 | 0.2 | -1032.3 | 1.3 | 0.3 | -2015.9 | 0.4 | 0.3 |
|  | Habitats + Water presence + Insect abundance | | -1888.0 | 26.4 | 0.0 | -1038.4 | 17.6 | 0.0 | -2019.4 | 11.5 | 0.0 |
|  | Habitats + Ruggedness + Insect abundance | | -1875.6 | 1.5 | 0.2 | -1031.2 | 3.1 | 0.1 | -2014.7 | 2.1 | 0.1 |
|  | Global |  | -1875.4 | 3.1 | 0.1 | -1030.8 | 4.4 | 0.1 | -2013.9 | 2.6 | 0.1 |
|  | Null |  | -1934.4 | 99.1 | 0.0 | -1090.1 | 100.9 | 0.0 | -2051.3 | 55.2 | 0.0 |
| July 15-31 | Habitats | | -2470.5 | 34.0 | 0.0 | -1444.4 | 39.1 | 0.0 | -2475.3 | 38.7 | 0.0 |
|  | Habitats + Water presence | | -2470.5 | 36.0 | 0.0 | -1441.3 | 34.8 | 0.0 | -2475.2 | 40.5 | 0.0 |
|  | Habitats + Ruggedness | | -2462.4 | 19.8 | 0.0 | -1426.3 | 4.9 | 0.1 | -2469.7 | 29.6 | 0.0 |
|  | Habitats + Insect abundance | | -2457.7 | 14.4 | 0.0 | -1439.1 | 34.5 | 0.0 | -2457.5 | 9.1 | 0.0 |
|  | Habitats + Water presence + Ruggedness | | -2462.4 | 21.8 | 0.0 | -1423.8 | 2.0 | 0.2 | -2469.7 | 31.6 | 0.0 |
|  | Habitats + Water presence + Insect abundance | | -2457.7 | 16.4 | 0.0 | -1435.9 | 30.2 | 0.0 | -2457.3 | 10.8 | 0.0 |
|  | Habitats + Ruggedness + Insect abundance | | **-2449.5** | **0.0** | **0.7** | -1422.3 | 2.9 | 0.1 | **-2451.9** | **0.0** | **0.7** |
|  | Global |  | -2449.5 | 2.0 | 0.3 | **-1419.8** | **0.0** | **0.6** | -2451.9 | 1.9 | 0.3 |
|  | Null |  | -2537.8 | 156.6 | 0.0 | -1559.8 | 257.9 | 0.0 | -2508.7 | 93.5 | 0.0 |

**S2 Table.** Candidate models tested to determine the effects of habitat types, water presence, terrain ruggedness, and insect abundance on the probability that female caribou were using a site for feeding. For each model, we provide the Log Likelihood (LL), delta AIC (∆ AIC), and model weight (***w_i_***).

**S2 Table continued.**

| **Period** | **Model** | | **Early summer (2017)** | | | **Intermediate summer (2016)** | | | **Late summer (2018)** | | |
| --- | --- | --- | --- | --- | --- | --- | --- | --- | --- | --- | --- |
|  |  |  | **LL** | **∆ AIC** | ***w_i_*** | **LL** | **∆ AIC** | ***w_i_*** | **LL** | **∆ AIC** | ***w_i_*** |
|  |  |  |  |  |  |  |  |  |  |  |  |
| August 1-14 | Habitats | | -1905.7 | 24.2 | 0.0 | -1328.9 | 35.1 | 0.0 | -2122.2 | 5.3 | 0.0 |
|  | Habitats + Water presence | | -1905.4 | 25.5 | 0.0 | -1328.7 | 36.7 | 0.0 | -2121.7 | 6.3 | 0.0 |
|  | Habitats + Ruggedness | | -1902.4 | 19.7 | 0.0 | **-1310.4** | **0.0** | **0.4** | -2122.1 | 7.1 | 0.0 |
|  | Habitats + Insect abundance | | -1892.2 | 3.2 | 0.1 | -1326.5 | 36.4 | 0.0 | **-2116.5** | **0.0** | **0.4** |
|  | Habitats + Water presence + Ruggedness | | -1902.1 | 21.0 | 0.0 | -1310.1 | 1.6 | 0.2 | -2121.6 | 8.1 | 0.0 |
|  | Habitats + Water presence + Insect abundance | | -1891.9 | 4.6 | 0.1 | -1326.3 | 37.9 | 0.0 | -2116.0 | 1.0 | 0.3 |
|  | Habitats + Ruggedness + Insect abundance | | **-1889.6** | **0.0** | **0.6** | -1307.8 | 1.0 | 0.3 | -2116.4 | 1.8 | 0.2 |
|  | Global |  | -1889.3 | 1.5 | 0.3 | -1307.6 | 2.6 | 0.1 | -2115.9 | 2.8 | 0.1 |
|  | Null |  | -2010.2 | 219.1 | 0.0 | -1397.7 | 158.7 | 0.0 | -2169.6 | 88.0 | 0.0 |
| August 15-31 | Habitats | | -2028.4 | 30.8 | 0.0 | -1639.4 | 21.2 | 0.0 | -2529.1 | 15.5 | 0.0 |
|  | Habitats + Water presence | | -2022.7 | 21.4 | 0.0 | -1638.6 | 21.5 | 0.0 | -2524.5 | 8.2 | 0.0 |
|  | Habitats + Ruggedness | | -2025.4 | 26.9 | 0.0 | -1629.8 | 4.1 | 0.1 | -2528.3 | 15.9 | 0.0 |
|  | Habitats + Insect abundance | | -2015.9 | 11.9 | 0.0 | -1634 | 16.4 | 0.0 | -2522.2 | 7.6 | 0.0 |
|  | Habitats + Water presence + Ruggedness | | -2019.7 | 17.4 | 0.0 | -1628.8 | 4.1 | 0.1 | -2523.7 | 8.7 | 0.0 |
|  | Habitats + Water presence + Insect abundance | | -2010.7 | 3.6 | 0.1 | -1633.1 | 16.6 | 0.0 | **-2517.3** | **0.0** | **0.5** |
|  | Habitats + Ruggedness + Insect abundance | | -2013.2 | 8.5 | 0.0 | -1624.8 | 0.1 | 0.4 | -2521.2 | 7.8 | 0.0 |
|  | Global |  | **-2007.9** | **0.0** | **0.8** | **-1623.8** | **0.0** | **0.5** | -2516.5 | 0.4 | 0.4 |
|  | Null |  | -2159.5 | 279.1 | 0.0 | -1696 | 120.4 | 0.0 | -259.0 | 127.3 | 0.0 |
