# Supplementary material for: Selection of summer feeding sites and food resources by female migratory caribou (Rangifer tarandus) determined using camera collars
Source: PLoS One. 2023 Nov 29;18(11):e0294846. doi: 10.1371/journal.pone.0294846 (PMC10686509; doi:10.1371/journal.pone.0294846)
Supplement: S3 Table — Each date represents the start of a two-week period. Percentages were calculated as the number of videos where a resource was consumed at least once divided by the total number of videos for that period. Multiple resource types could be consumed in one video. (DOCX) [file pone.0294846.s004.docx]

|  |  | **Percentage of videos** | | | | | |
| --- | --- | --- | --- | --- | --- | --- | --- |
| **Summer timing and resource type** | | **June** | | **July** | | **August** | |
|  |  | **1** | **15** | **1** | **15** | **1** | **15** |
| *Early summer transition (2017)* | | | | | | | |
| Lichens |  | 41 | 16 | 8 | 22 | 29 | 32 |
| Graminoids | | 30 | 43 | 48 | 34 | 17 | 16 |
| Other shrubs | | 3 | 3 | 1 | 4 | 6 | 8 |
| Low vegetation | | 24 | 30 | 33 | 25 | 23 | 23 |
| Other herbaceous | | 0 | 2 | 4 | 1 | 2 | 1 |
| Birches | | 2 | 5 | 3 | 11 | 18 | 12 |
| Willows |  | 0 | 1 | 1 | 2 | 3 | 4 |
| Mosses |  | 1 | 0 | 0 | 0 | 0 | 0 |
| Mushrooms | | 0 | 0 | 0 | 0 | 3 | 3 |
|  | |  |  |  |  |  |  |
| *Intermediate summer transition (2016)* | | | | | | | |
| Lichens |  | 55 | 46 | 19 | 17 | 31 | 38 |
| Graminoids | | 14 | 24 | 36 | 39 | 23 | 7 |
| Other shrubs | | 3 | 2 | 2 | 2 | 5 | 7 |
| Low vegetation | | 26 | 27 | 35 | 31 | 22 | 12 |
| Other herbaceous | | 0 | 0 | 5 | 6 | 5 | 5 |
| Birches | | 0 | 0 | 2 | 1 | 13 | 23 |
| Willows |  | 0 | 0 | 1 | 2 | 1 | 5 |
| Mosses |  | 2 | 2 | 0 | 1 | 0 | 0 |
| Mushrooms | | 0 | 0 | 0 | 0 | 0 | 3 |
|  | |  |  |  |  |  |  |
| *Late summer transition (2018)* | | | | | | | |
| Lichens |  | 63 | 63 | 37 | 16 | 25 | 25 |
| Graminoids | | 8 | 16 | 30 | 37 | 29 | 16 |
| Other shrubs | | 1 | 2 | 1 | 1 | 1 | 1 |
| Low vegetation | | 26 | 18 | 24 | 34 | 30 | 19 |
| Other herbaceous | | 0 | 0 | 1 | 2 | 2 | 3 |
| Birches | | 0 | 0 | 6 | 6 | 11 | 31 |
| Willows |  | 0 | 0 | 0 | 1 | 2 | 5 |
| Mosses |  | 1 | 2 | 1 | 1 | 0 | 0 |
| Mushrooms | | 0 | 0 | 0 | 0 | 0 | 1 |

**S3 Table.** Percentages of videos where a food resource type was observed as consumed. Each date represents the start of a two-week period. Percentages were calculated with the number of videos where a resource was consumed at least once and the total number of videos for that period. Multiple resource types could be consumed in one video.
