# Supplementary material for: Selection of summer feeding sites and food resources by female migratory caribou (Rangifer tarandus) determined using camera collars
Source: PLoS One. 2023 Nov 29;18(11):e0294846. doi: 10.1371/journal.pone.0294846 (PMC10686509; doi:10.1371/journal.pone.0294846)
Supplement: S4 Table — Each date represents the start of a two-week period. Percentages were calculated with the number of videos where a habitat was seen as used and the total number of videos for that period. (DOCX) [file pone.0294846.s005.docx]

|  |  | **Percentages of videos** | | | | | |
| --- | --- | --- | --- | --- | --- | --- | --- |
| **Summer timing and habitat type** | | **June** | | **July** | | **August** | |
|  |  | **1** | **15** | **1** | **15** | **1** | **15** |
| *Early summer transition (2017)* | | | | | | | |
| Ericaceous tundra | | 25 | 14 | 6 | 16 | 14 | 15 |
| Tundra | | 54 | 64 | 76 | 41 | 18 | 14 |
| Tundra with shrubs | | 8 | 11 | 7 | 24 | 40 | 44 |
| Wetland | | 11 | 9 | 7 | 7 | 1 | 1 |
| Wetland with shrubs | | 0 | 1 | 0 | 2 | 1 | 2 |
| Shrubland | | 1 | 2 | 3 | 9 | 25 | 20 |
| Rocky ground | | 1 | 0 | 0 | 0 | 0 | 0 |
| Snowy ground | | 1 | 0 | 0 | 0 | 0 | 0 |
| Taiga | | 0 | 0 | 0 | 0 | 0 | 4 |
| *Intermediate summer transition (2016)* | | | | | | | |
| Ericaceous tundra | | 24 | 28 | 20 | 19 | 25 | 10 |
| Tundra | | 51 | 49 | 49 | 53 | 34 | 5 |
| Tundra with shrubs | | 6 | 2 | 5 | 5 | 13 | 18 |
| Wetland | | 8 | 19 | 23 | 20 | 4 | 2 |
| Wetland with shrubs | | 0 | 0 | 1 | 1 | 2 | 2 |
| Shrubland | | 2 | 0 | 2 | 1 | 20 | 58 |
| Rocky ground | | 2 | 2 | 0 | 0 | 1 | 0 |
| Snowy ground | | 6 | 0 | 0 | 0 | 0 | 0 |
| Taiga | | 0 | 0 | 0 | 0 | 0 | 5 |
| *Late summer transition (2018)* | | | | | | | |
| Ericaceous tundra | | 44 | 43 | 29 | 19 | 22 | 8 |
| Tundra | | 30 | 28 | 32 | 56 | 40 | 11 |
| Tundra with shrubs | | 12 | 9 | 15 | 13 | 19 | 36 |
| Wetland | | 4 | 11 | 16 | 7 | 7 | 2 |
| Wetland with shrubs | | 0 | 1 | 3 | 2 | 2 | 4 |
| Shrubland | | 5 | 6 | 4 | 4 | 9 | 36 |
| Rocky ground | | 1 | 2 | 1 | 1 | 1 | 0 |
| Snowy ground | | 5 | 1 | 0 | 0 | 0 | 0 |
| Taiga | | 0 | 0 | 0 | 0 | 0 | 3 |

**Table S4.** **Percentages of videos where a habitat type was used as a feeding site at least once by female migratory caribou of the RFH, in northern Québec, Canada**. Each date represents the start of a two-week period. Percentages were calculated with the number of videos where a habitat was seen as used and the total number of videos for that period.
