# Supplementary material for: Selection of summer feeding sites and food resources by female migratory caribou (Rangifer tarandus) determined using camera collars
Source: PLoS One. 2023 Nov 29;18(11):e0294846. doi: 10.1371/journal.pone.0294846 (PMC10686509; doi:10.1371/journal.pone.0294846)
Supplement: S6 Table — For description of each habitat, resource types and other variables, please see main article. We considered a site used when an individual foraged at least once during a video. On the contrary, if an individual was exhibiting any other behavior (e.g., resting, walking, vigilance) without foraging, we considered this site to be unused. (DOCX) [file pone.0294846.s007.docx]

**S6 Table. Set of examples videos recorded by camera collars on migratory caribou of the Rivière-aux-Feuilles herd in summer in northern Quebec, Canada.** For description of each habitat, resource types and other variables, please see main article. We considered a site used when an individual foraged at least once during a video. On the contrary, if an individual was exhibiting any other behavior (e.g., resting, walking, vigilance) without foraging, we considered this site to be unused.

| **URL** | **Used** | **Habitat** | **Resource(s) consumed** | **Resource(s) unconsumed** | **Water presence** | **Rugged** | **Insects** |
| --- | --- | --- | --- | --- | --- | --- | --- |
| <https://youtu.be/VIQybLD02iM> | no | Tundra with shrubs | n/a | Graminoids, low vegetation, birches, mosses | no | no | absent |
| <https://youtu.be/oRQg3cOn7FA> | no | Wetland with shrubs | n/a | Graminoids, low vegetation, birches, mosses, lichens | no | no | absent |
| <https://youtu.be/ojHC74mOJaM> | no | Rocky ground | n/a | Graminoids, low vegetation, birches, mosses, lichens | no | yes | absent |
| <https://youtu.be/K7xciKeifV8> | no | Tundra | n/a | Graminoids, low vegetation, mosses, lichens | no | no | absent |
| <https://youtu.be/aMfTQ0RPpM0> | no | Snowy Ground | n/a | n/a | no | no | absent |
| <https://youtu.be/K8mR9k20BvU> | no | Tundra | n/a | Graminoids, lichens, mosses | no | no | high |
| <https://youtu.be/eX3d09rX9fU> | no | Rocky ground | n/a | n/a | yes | no | absent |
| <https://youtu.be/lPMOrwGDtMU> | no | Wetland | n/a | Graminoids | no | no | high |
| <https://youtu.be/4wtepYo2is8> | yes | Ericaceous tundra | Graminoids, low vegetation | Lichens, other shrubs, other herbaceous, mosses | no | no | absent |
| <https://youtu.be/Ca-yTHPVSnk> | yes | Taiga | Lichens, graminoids | Low vegetation, mosses, birches | no | no | absent |
| <https://youtu.be/sdKhhED9aKc> | yes | Shrubland | Mushrooms | Birches, other shrubs, low vegetation, graminoids, lichens, mosses | no | no | absent |
| <https://youtu.be/ZkK0DOeHHgc> | yes | Tundra | Other herbaceous, graminoids | Low vegetation, mosses | no | no | absent |
| <https://youtu.be/lujjHaM_1Qk> | yes | Wetland | Graminoids | n/a | no | no | absent |
| <https://youtu.be/1MeHcygMU-8> | yes | Shrubland | Willows | Birches, graminoids | no | no | absent |
