# Supplementary figures and images for: Selection of summer feeding sites and food resources by female migratory caribou (Rangifer tarandus) determined using camera collars
Source: PLoS One. 2023 Nov 29;18(11):e0294846. doi: 10.1371/journal.pone.0294846 (PMC10686509; doi:10.1371/journal.pone.0294846)

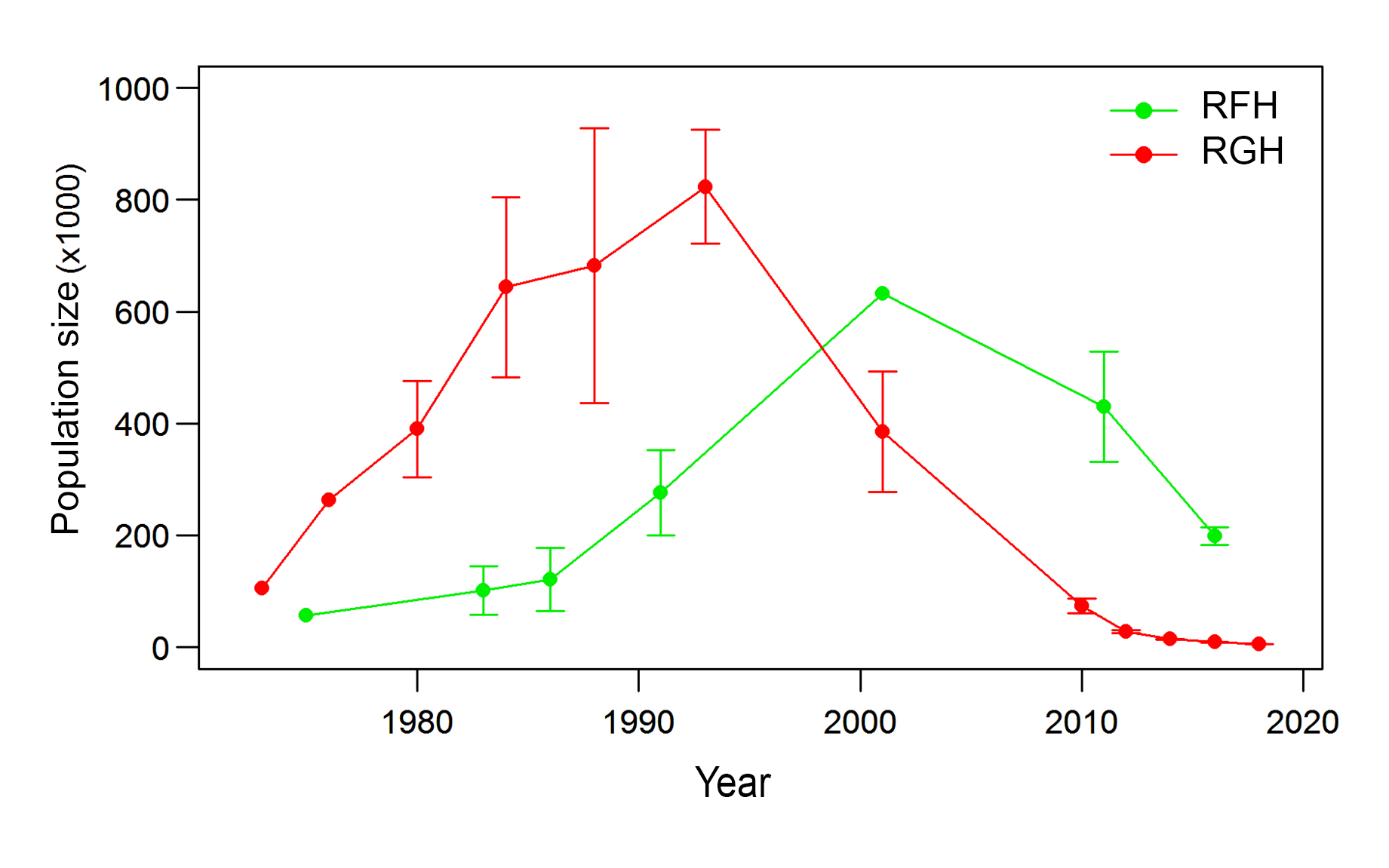

Supplement: S1 Fig — Points without a confidence interval represents a minimal count and had no error associated to it [20]. (TIF) [file pone.0294846.s008.tif]
